# Nitrogen fluxes at the root-soil interface show a mismatch of nitrogen fertilizer supply and sugarcane root uptake capacity

**DOI:** 10.1038/srep15727

**Published:** 2015-10-26

**Authors:** Richard Brackin, Torgny Näsholm, Nicole Robinson, Stéphane Guillou, Kerry Vinall, Prakash Lakshmanan, Susanne Schmidt, Erich Inselsbacher

**Affiliations:** 1 School of Agriculture and Food Sciences, The University of Queensland, QLD, 4072, Australia; 2 Department of Forest Ecology and Management, Swedish University of Agricultural Sciences, SE-901 83 Umeå, Sweden; 3 Department of Forest Genetics and Plant Physiology, Umeå Plant Science Center, Swedish University of Agricultural Sciences, SE-901 83 Umeå, Sweden; 4 Sugar Research Australia, 50 Meiers Road, Indooroopilly, QLD 4068, Australia; 5 University of Vienna, Department of Geography and Regional Research, Vienna, AT-1090, Austria

## Abstract

Globally only ≈50% of applied nitrogen (N) fertilizer is captured by crops, and the remainder can cause pollution *via* runoff and gaseous emissions. Synchronizing soil N supply and crop demand will address this problem, however current soil analysis methods provide little insight into delivery and acquisition of N forms by roots. We used microdialysis, a novel technique for *in situ* quantification of soil nutrient fluxes, to measure N fluxes in sugarcane cropping soils receiving different fertilizer regimes, and compare these with N uptake capacities of sugarcane roots. We show that in fertilized sugarcane soils, fluxes of inorganic N exceed the uptake capacities of sugarcane roots by several orders of magnitude. Contrary, fluxes of organic N closely matched roots’ uptake capacity. These results indicate root uptake capacity constrains plant acquisition of inorganic N. This mismatch between soil N supply and root N uptake capacity is a likely key driver for low N efficiency in the studied crop system. Our results also suggest that (i) the relative contribution of inorganic N for plant nutrition may be overestimated when relying on soil extracts as indicators for root-available N, and (ii) organic N may contribute more to crop N supply than is currently assumed.

Nitrogen (N) uptake by crops is a key constituent of the global N cycle, as N captured by roots has a markedly different fate than N remaining in the soil. The success or failure of plants to capture N in the root zone has implications not only for crop growth and yield, but also for losses of reactive N from agro-ecosystems *via* leaching, runoff and emission as nitrogenous gases. Globally, in excess of 100 Tg of N are applied annually to crops^1^,^2^ of which ~55 Tg N y^−1^ is captured by crops or remains in the soil, and ~45 Tg N y^−1^ are estimated to be lost to the environment^2^,^3^. This inefficiency is of global concern^4^, and requires innovation based on improved understanding of how N is transformed in soils, and how N transformations affect N uptake by crops^5^,^6^.

Nitrate and ammonium (inorganic N) are considered to be the main N sources for crops, largely due to their prevalence in agricultural soils^7^. Inorganic N forms are direct precursors for gaseous N, and nitrate is prone to leaching from soil^6^. Plants also take up and metabolize a wide range of organic N forms present in soil, including amino acids^8^, peptides^9^,^10^,^11^, proteins^12^ and quaternary ammonium compounds^13^. Amino acid uptake has been demonstrated in every plant species studied, including *Arabidopsis thaliana*^14^ and crops such as barley and sugarcane^15^,^16^. The quantitative importance of organic N to the plant N budget has not been established in any ecosystem, but the ubiquitous capacity of plants to absorb both inorganic and organic N suggests the composition of the plant-available N pool in soil should exert a strong influence on the form of N acquired by plants.

Determining which N forms are available for and ultimately taken up by crops remains a challenge. Destructive soil sampling and subsequent processing introduces artefacts such that the size and composition of the N pools deviate from those *in situ*^17^,^18^, suggesting that the widespread use of soil extracts as proxy for N availability to plants may be flawed. This conflict is illustrated when comparing information obtained with conventional excavation-sieving-extraction techniques and low-disturbance *in situ* microdialysis^19^. A further drawback of conventional soil sampling techniques is that they do not provide information on flux rates of N compounds in soils, although flux rates, rather than bulk soil N concentrations, are critical drivers of root uptake^20^,^21^,^22^. Microdialysis induces diffusive fluxes in soils, and at high perfusate flow rates, close to maximum diffusive fluxes of external N compounds are obtained^23^. While the small size of microdialysis probes means that a smaller volume of soil can be sampled, relative to that sampled via soil extracts, it also allows the study of N dynamics in soil microsites. Because microdialysis probes share important features with roots, such as their small size, as well as their shape and mode of action^24^, we propose that it should be possible to compare soil N delivery and root uptake rates. Matching soil N supply to the crops’ N demand is a key objective for improving the nutrient use efficiency of cropping systems^5^,^25^,^26^,^27^.

Globally, sugarcane cropping systems have high N application rates, and large environmental N losses^28^,^29^. We examined N fluxes in sugarcane soils and compared these with N pools derived from soil extracts. Fluxes and pools of inorganic and low molecular mass organic N (amino acids) were quantified in soils receiving either no fertilizer, predominantly organic fertilizer (sugar mill waste and crop residues), or synthetic (urea) fertilizer. Independent N uptake experiments with excised roots of field-grown sugarcane allowed calculation of maximum N uptake per unit root surface area per hour (I_max_), allowing direct comparison between induced soil fluxes (calculated per unit microdialysis-probe surface area and time) and maximum root uptake flux capacity for inorganic and organic N (per unit root surface area and time). With this novel approach, we aimed to discern the relationship between soil N delivery *via* diffusive fluxes and the roots’ ability to acquire N. Root uptake capacity was quantified under conditions of reduced microbial competition, so the results represent the maximum possible N acquisition rate of roots. By contrast, *in situ* microdialysis probes acquire N in competition with soil biota, representing realistic rates of N arrival at the root surface in soil.

## Results and Discussion

**Figure 1:**
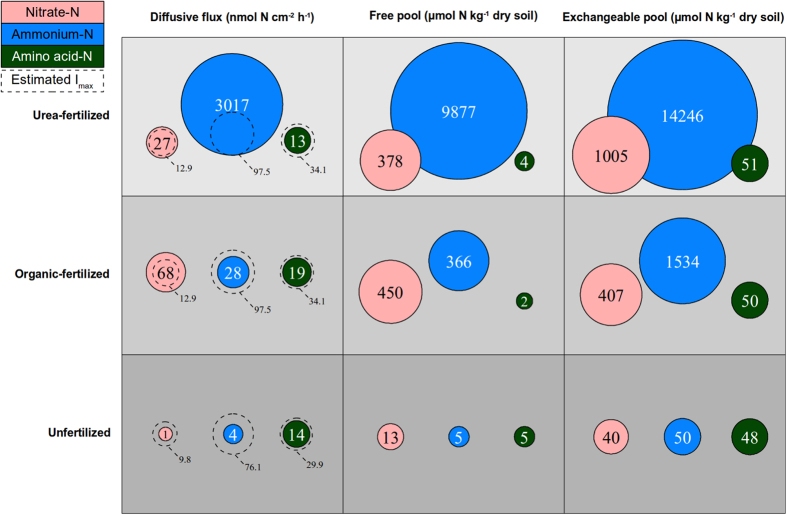
Fluxes and concentrations of nitrate-N (pink), ammonium-N (blue) and amino acid-N (green) in the three soils under sugarcane. Circle area is determined by the square root of pool size or flux. Mean pool size of 40 replicate flux measurements or soil extractions is shown inside each circle. Estimated I_max_ values (maximum estimated root intake rate) for sugarcane root uptake under fertilized and unfertilized conditions (nmol N cm^−2^ h^−1^ for nitrate, ammonium and glycine) are represented by dotted lines for comparison with fluxes. Free N pools (as determined by water extracts) and exchangeable N pools (as determined by KCl extracts) are shown in μmol N kg^−1^ dry soil. For more detail and statistics see Table S1.

Ammonium fluxes ranged from 4.1 to 3017 nmol N cm^−2^ h^−1^ in unfertilized and urea-fertilized soil, respectively (Fig. 1, Supplementary Table 1). Nitrate fluxes spanned from 1.3 to 68 nmol N cm^−2^ h^−1^ in unfertilized and organic-fertilized soil, respectively. Overall, fluxes of amino acids were more similar across soils than fluxes of inorganic N and significantly (P < 0.003) higher in organic-fertilized soil (19 nmol N cm^−2^ h^−1^) than in urea-fertilized and unfertilized soils (13 and 14 nmol N cm^−2^ h^−1^, respectively; Fig. 1, Supplementary Table 1).

**Figure 2:**
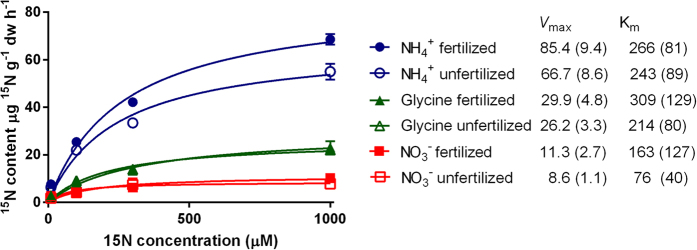
Fitted Michaelis-Menten curves of incorporation of ^15^N-labelled ammonium (circles), glycine (triangles) or nitrate (squares) by excised roots from plants grown in fertilized (solid symbols) or unfertilized (hollow symbols) soils. Roots were incubated in an equimolar solution of the three N sources with one N source ^15^N-labelled and two N sources unlabeled. Data represents averages ^15^N incorporation for each N source across eight replicate plants at four time points. Error bars are standard error of the mean. Estimated *V*_max_ values for each curve are inset.

**Figure 3:**
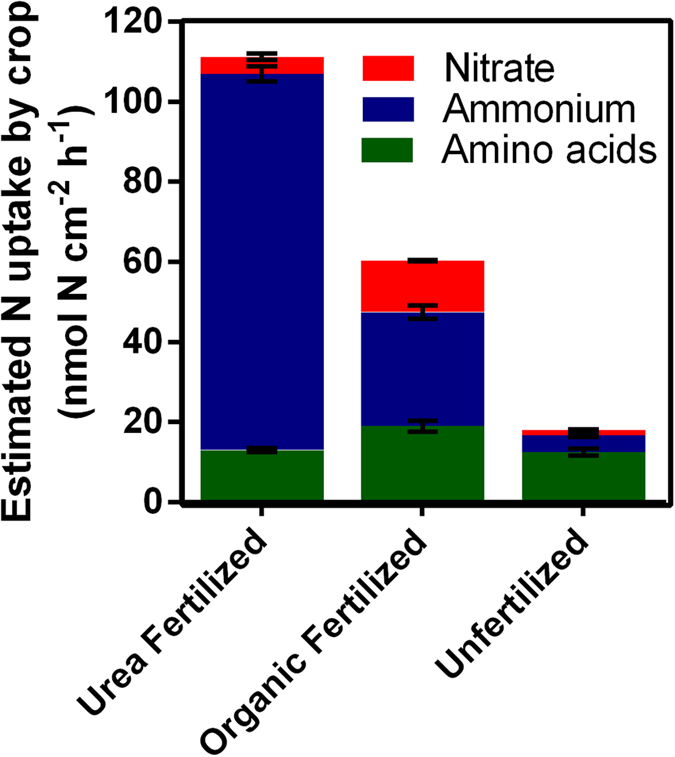
Estimated plant N uptake (per unit root surface area) under three soil treatments, based on soil N flux rates and root uptake capacity (I_max_). These estimates have been calculated using root I_max_ values as a cut-off (I_max_ value is used where soil fluxes exceed this value). Where soil fluxes are lower than I_max_, soil flux values have been used. This data has been calculated from 40 replicate measurements for each treatment. Error bars are standard error of the mean.

Plant N preferences can be determined with excised or intact roots^30^,^31^. Excised root experiments are easier to conduct, but may underestimate uptake of ions such as nitrate that are influenced by mass flow^31^,^32^. We supplied excised sugarcane roots simultaneously with reciprocally isotopically labelled ^15/14^N sources (nitrate, ammonium, and representative amino acid glycine) at concentrations ranging from 0–1000 μM to determine uptake kinetics (Figs 2 and 3). The concentrations were chosen to target the operational range of high-affinity transport systems^33^. We additionally conducted experiments on intact sugarcane roots *in situ* to validate the data obtained from excised roots. Due to difficulties associated with excavating enough undamaged intact roots, the intact root experiments were conducted only at a single nutrient concentration. Intact and excised root uptake experiments produced similar N uptake proportions (Supplementary Fig. 1). Uptake of nitrate was not higher in attached roots, indicating that sink strength was not a major factor in low nitrate uptake in this experiment (Supplementary Fig. 1; Supplementary methods and materials). Uptake of all three N sources was ~30% higher in excised than attached roots (Supplementary Fig. 1), suggesting the possibility that excised root experiments may have over-estimated N uptake rates: if this is the case, then I_max_ values would be smaller than those reported here by ~30%. The I_max_ values from excised roots which we have used therefore represent a conservative estimate of the mismatch between soil fluxes and root uptake. Uptake kinetics differed between plants in fertilized and unfertilized soils, but no significant differences were observed between sampling time points at different times of the growing season, and annual average data is presented (Fig. 2). Excised roots exhibited maximum uptake rates per unit weight (V_max_) of 85.4, 29.9 and 11.3 μmol g^−1^ dry weight h^−1^ for ammonium, glycine, and nitrate, respectively in fertilized plants, and 66.7, 26.2 and 8.6 μmol g^−1^ dry weight h^−1^ for unfertilized plants (Fig. 2). K_m_ values were calculated (Fig. 2), and together with an average root surface area-to-weight ratio of 876 cm^2^ g^−1^ dry weight, we estimated I_max_ values to be 97.5 (with a standard error of 10.7), 34.1 (5.1) and 12.9 (3.1) nmol N cm^−2^ h^−1^ for ammonium, glycine and nitrate, respectively in fertilized plants; and 76.1 (9.8), 29.9 (3.8) and 9.8 (1.3) nmol N cm^−2^ h^−1^ in unfertilized plants (Fig. 1). While uptake experiments were conducted in a different location to the soil N flux assessment, we are confident that results from the two sites can be directly compared. We have strong accumulated evidence that sugarcane N preferences and N uptake rates and proportions are reasonably consistent across a wide range of variables, including time of year, crop age, N fertilizer rate, location, soil type and sugarcane genotype (Fig. 2, Supplementary Fig. 1)^28^,^34^. Uptake rates and therefore I_max_ will vary by a small percentage based on the factors listed above, however this will be very minor compared to size of soil N fluxes which are orders of magnitude greater than the respective root uptake capacities.

Our calculations show that fluxes of ammonium and nitrate in the of urea-fertilized soil exceed the root’s estimated uptake capacity, I_max_, at ~3094 and ~209% of I_max_, respectively. Nitrate fluxes exceeded I_max_ in organic fertilized soil, representing ~527% of I_max_ (Fig. 1). This disparity between fluxes of inorganic N and I_max_ emphasizes the mismatch between N supply and N acquisition ability at the soil-root interface. This mismatch is likely to constitute a key factor for the low N use efficiency that characterizes many high-productivity crop systems, including sugarcane, which receive high rates of synthetic N fertilizer^28^,^35^,^36^. Soil N fluxes beyond the level of root I_max_ are effectively unavailable to the plant, rendering the excess N vulnerable to loss.

Considerable N losses characterize cropping systems worldwide^5^, including those receiving only organic fertilizers^37^. Release of N from organic materials is typically slower than N release from synthetic fertilizers, due to the need for multiple stages of microbial decomposition^38^. Nevertheless, organic-fertilized systems lose N, particularly if high rates of organic matter are applied in order to compensate for low initial availability of inorganic N in the organic matter^37^,^39^,^40^. The organic-fertilized sugarcane field in our study received ~380 kg N ha^−1^ over the past 12 months, compared to 155 kg N ha^−1^ applied to the urea fertilized field. At the time of measurement, both sites showed a significant mismatch in soil N supply and crop N acquisition.

In unfertilized soil, diffusive fluxes of ammonium and nitrate were well below the estimated I_max_ (~10% of I_max_, Fig. 1). Glycine was one of the most prominent amino acids in the diffusive fluxes (data not shown), and was used here to approximate root uptake capacity of amino acids. In contrast to inorganic N, soil organic N fluxes closely matched root uptake capacity in all tested soils, ranging from 38–56% of I_max_.

The current view that (non-N_2_ fixing) crops depend on inorganic sources for their N nutrition is based on three observations and assumptions: (i) easily accessible organic N (such as amino acids) is not an important component of the soil solution because concentrations of inorganic N generally exceed organic N by several orders of magnitude, (ii) plants are inferior competitors for organic N in the presence of soil microbes, and (iii) crops in N limiting soils respond to additions of inorganic N fertilizer^7^,^8^,^41^. The view that inorganic N is the main source of N for crops is founded on experiments that show soil microbes compete effectively with roots for amino acids. Such experiments typically spike soil with ^15^N^13^C-labelled amino acids at high concentration (1–75 mM) in single applications, followed by quantification of the isotope label in microbes and plant tissues after 2–48 hours^42^,^43^,^44^. Such experiments, including isotopic analysis of microbe and plant tissues by NanoSIMS^45^, as well as the determination of uptake kinetics of amino acids by microbes and roots^46^, suggest that plants acquire only a small fraction of the pulse-supplied organic N, while most is acquired by soil microbes. However, experiments using a single application of amino acids are likely to favor uptake by microbes due to successful competition for a short term N pulse^46^,^47^.

Avoiding artificial increases of the soil amino acid pool, microdialysis probes acquire N in competition with soil microbes. We argue that the presence of amino acids in dialysates indicates their availability at the root surface. Low concentrations, but high turnover rates and continuous availability appears to be the typical presence of amino acids in the soluble N pool of soils^41^,^48^. This indicates that even though plants may be inferior competitors for a single spike of organic N, over time they may acquire a substantial proportion of their N demand from organic N in undisturbed soil^41^,^47^.

**Figure 4:**
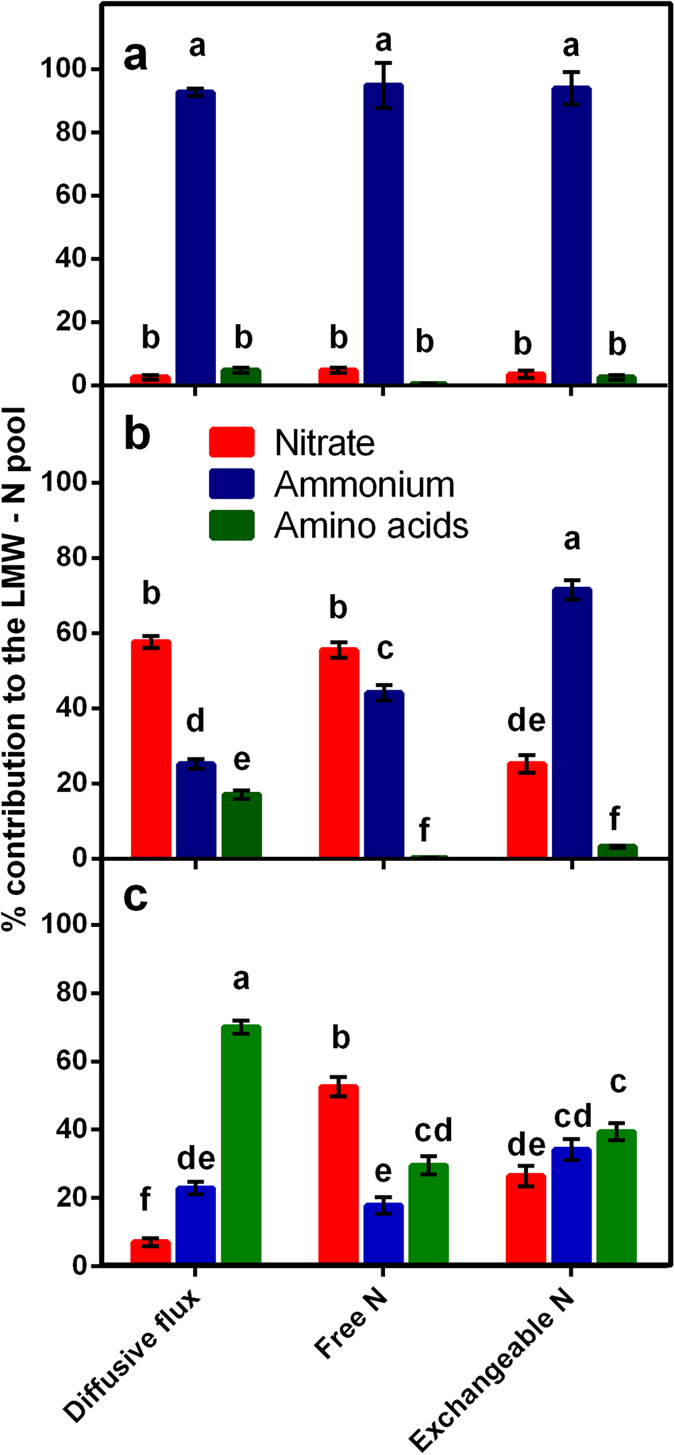
Contribution of nitrate, ammonium and amino acids to plant- available N, as obtained by diffusive fluxes, free N (as determined by water extracts) and exchangeable N pools (as determined by KCl extracts); in urea-fertilized (**a**), organic fertilized (**b**) and unfertilized soil (**c**). Data are derived from 40 replicate probes and corresponding soil samples at each site. Error bars are standard error of the mean, lower case letters represent significant differences (*P* < 0.05) within each soil.

Here we tested the assumption that the observed dominance of inorganic N in the soil solution precludes use of organic N by crops by linking soil diffusive fluxes to root I_max_ values. We argue that sugarcane roots have the capacity to use only a small fraction of the inorganic N delivered *via* diffusive flux after fertilizer application, but most or all of the organic N flux. This suggests that the contribution of organic N such as amino acids may be substantial even in soils fertilized with urea. For the purposes of a simplistic model, we assumed that plant N acquisition occurs at the rate of I_max_ when soil fluxes exceed this rate, and at soil flux rates when these are lower than I_max_. This allows calculation of an estimated N acquisition profile for sugarcane (Fig. 3). This model indicates that sugarcane plants in the urea-fertilized soil take up ammonium > amino acids > nitrate at relative proportions of 84, 12 and 4%. By contrast, if the 95 and 5% dominance of ammonium and nitrate in urea-fertilized soil N pools as measured by extracts (Fig. 4) is the assumed indicator of plant N use, the contribution of inorganic N is greatly overestimated. Similarly in the organic fertilized soil, our model indicates uptake of ammonium > amino acids > nitrate at proportions of 46, 31 and 23%, despite a free N pool (as determined by water extracts) with proportions of 44, 0.3 and 56%, and an exchangeable pool (as determined by KCl extracts) of 72, 3 and 25% (Figs 1 and 4). This gives a strong indication that soil N pools may be a poor indicator of plant N supply.

The extent to which different N sources contribute to plant N nutrition under conditions of high N flux would, however, depend on how the different N sources interact during uptake. It is established that ammonium exerts a strong inhibitory effect on nitrate uptake^49^. There are few reports on the interactions between inorganic and organic N uptake. Studies that have targeted the interaction between inorganic N and amino acid uptake suggest amino acids may affect inorganic N uptake negatively, but inorganic N does not inhibit uptake of amino acids^50^,^51^,^52^.

While the contribution of organic N to crop N budgets remains unclear, it is becoming apparent that amino acids and peptides can represent a significant proportion of the soil N pools in agricultural soils^11^,^41^,^43^,^53^,^54^. Boreal forest and sub-tropical sugarcane soils represent opposite ends of the N availability spectrum, and the similar prevalence of amino acids in N fluxes in these soils contradicts expectations of contrasting relative dominance of organic N cf.^47^. Globally, sugarcane soils typically receive high N fertilizer rates in the form of granular urea that is applied as one dose during early crop growth^55^. This practice results in ≈3 months of very high soil inorganic N concentrations (the two fertilized soils sampled here are representative of this period) followed by an extended period of low inorganic N concentrations until harvest at 12–14 months^28^,^41^,^55^. The unfertilized soil here is broadly representative of N availability over the final ~9 months of the sugarcane growing season as the size and relative composition of exchangeable N pools mirror those of soil during the later growth phase^41^. The results here indicate that during the ~9 months when sugarcane soils have a low availability of inorganic N, amino acids are a prominent N source that can make a considerable contribution to crop N demand. The prolonged growth of sugarcane necessitates that ≈50% of shoot N is acquired during the period of low soil N availability^28^, and our results suggest that amino acids may comprise a substantial fraction of N acquisition during this time with contributions by amino acids > ammonium >nitrate of 70 > 23 > 7% to the plant N budget (Figs 3 and 4). While measurements here have only assessed soil and roots from the top 20 mm of the soil profile due to the small size of microdialysis probes, soil extract results in this study are within the range determined across the crop rooting zone (0–20 cm) in previous studies^41^,^55^, indicating that these measurements are likely to broadly reflect availability to the crop. Future research will include measurements at a range of depths via sequential excavation of topsoil.

Current sugarcane cropping systems have poor N use efficiency because inorganic N accumulates in high concentrations, which exceed the N acquisition ability of crops and soil biota. We hypothesize that the mismatch between soil N supply and sugarcane N acquisition ability is likely to be similar for other crops, however further study of cropping systems with a focus on soil N fluxes is required. Here we show a new approach for examining N relations in soil in context of crop N physiology, which provides a new avenue towards tailoring N fertilizer supply to match the specific uptake abilities and N demand of crops over the growth cycle.

## Materials and Methods

### Study site

Nutrient fluxes were determined at a sugarcane farm (27°46′41.79′′S, 153°19′37.33′′E) near Jacob’s Well, Queensland, Australia. Soil properties and climate are shown in Supplementary Table 2. Three adjacent fields received different fertilizer additions – urea fertilizer, organic fertilizer and no fertilizer. The ‘urea fertilized’ field was supplied with 135 kg N ha^−1^ as urea 10 days prior to sampling. Rain and irrigation (~2.3 mm) occurred between fertilizer application and soil sampling. The field receiving organic fertilizer had residual plant litter from the previous rotation of soybeans. Additionally, 100 t fresh weight ha^−1^ of sugarcane industry waste (mill mud’), containing approximately 330 kg N ha^−1^ of which over 90% is organic N^56^, had been applied 12 months previously. Information on agronomic practices is summarized in Table 1. Nitrogen additions from organic forms were calculated using farm estimations of input tonnage and published %N values for the input forms^56^,^57^.

### Determination of N fluxes and pools

Five sub-sites were sampled from each field. Eight samples were collected at each of the five sites, resulting in 40 samples per field. The five sub-sites were each separated by at least 10 m and no two sub-sites were in the same row of cane. Our five sub-sites were distributed across an area of ~300 m^2^ in each field.

Microdialysis membrane calibration and soil sampling was done as described previously^23^. Briefly, polyarylethersulphone probes (CMA 20, CMA Microdialysis AB, Kista, Sweden; 10 mm long, 500 μm outer diameter and 400 μm inner diameter) were inserted to approximately 15 mm depth. Perfusate (high-purity deionized water) was pumped through the system by syringe infusion pumps (CMA 400) at a flow rate of 5 μl min^−1^and samples were collected for 1 h sampling time in refrigerated micro-fraction collectors (CMA 470).

Diffusive fluxes from the soil are calculated as per Inselsbacher and Näsholm^19^, and expressed as nmol cm^−2^ h^−1^.

Samples were stored at ~6 °C for <10 h and then frozen (−20 °C) until analysis of N compounds. After microdialysis collection, soil was sampled to ~2 cm depth in the immediate vicinity of the probe, to provide the closest possible approximation to the estimated area sampled by the probe. Soil was stored in 50 ml centrifuge tubes at ~20 °C, and subsequently extracted using deionized water (to determine free N pools) or 1.5 M KCl (to determine total exchangeable N pools, which incorporate the free N pool) within 24 h as per Holst *et al.*^41^.

### Root uptake kinetics

Plant N uptake kinetics were assessed at a commercial sugarcane farm (25°00′11′′S, 152°19′08′′E) near Bundaberg, Queensland, Australia. Nitrogen source preference of excised roots was assessed at four time points across the growing season: December, January, March and May. Ambient temperature during the experiments was approximately 25–30 °C in December and January, 20–27 °C in March and 18–24 °C in May. Analysis was performed on roots from both fertilized (100 kg N ha^−1^, supplied as urea) and unfertilized (0 kg N ha^−1^) plants.

Roots were harvested by manually excavating soil and roots adjacent to four sugarcane plants. Roots were washed thoroughly with water and stored in zip-lock plastic bags at ambient temperature prior to incubation experiments, which were conducted within four hours. Roots were cut into 2 cm sections. These were divided into 12 sub-samples, and each sub-sample was placed in a vial containing 30 mL of N solution. Each solution consisted of an equimolar mixture of ammonium (as (NH_4_)_2_SO_4_), nitrate (as KNO_3_), and glycine, with one of the N sources labelled with ^15^N (98–99 atom % excess) and the other two N sources at natural abundance level. Glycine was chosen as it is one of the most prominent amino acids in the current investigation. Each treatment consisted of four N concentrations: 10, 100, 300, or 1000 μM ^15^N-labelled N source (30, 300, 900, or 3000 μM total N in solution). The concentrations were chosen to target the operational range of high-affinity transport systems^33^. Each solution was prepared with 100 μM CaSO_4_ to maintain cell membrane integrity. Vials were agitated gently on a shaker for 30 min at ambient temperature. The N solution was then replaced with 60 mL of 10 mM KCl and roots were agitated for a further 10 min to remove unincorporated N. Potassium chloride was replaced with 60 mL water and roots were shaken for 5 min before being removed from solution. Root samples were dried at 55 °C, then ground to a fine powder via ball mill (Retsch MM2).

### Analyses

Nitrate concentrations in soil were analyzed using vanadium chloride and subsequent Griess reaction^58^. Amino acids and NH_4_^+^ were analyzed via UPLC as per Holst *et al.*^41^.

Plant Samples were analyzed for ^15^N by a PDZ Europa ANCA-GSL elemental analyzer interfaced to a PDZ Europa 20–20 isotope ratio mass spectrometer (Sercon Ltd., Cheshire, UK). Michaelis-Menten curves were fitted to the ^15^N data, and the estimated *V*_max_ values were calculated using Graphpad Prism 6.02 (GraphPad Software, SanDiego, CA, USA). Estimated *I*_max_ values were calculated using *V*_max_ values, and average sugarcane root surface area per gram dry tissue weight (876 cm^2^ g^−1^ dw) as determined by WinRHIZO software (v. 2007d, Regent Instruments, Quebec, Canada).

Data were analyzed using one-way ANOVA with Tukey’s Post-hoc test (Statistica version 10, StatSoft Inc, Tulsa, USA). Where necessary, data were arcsine or log_10_ transformed to meet assumptions of ANOVA analysis.

## Additional Information

**How to cite this article**: Brackin, R. *et al.* Nitrogen fluxes at the root-soil interface show a mismatch of nitrogen fertilizer supply and sugarcane root uptake capacity. *Sci. Rep.* **5**, 15727; doi: 10.1038/srep15727 (2015).

## Supplementary Material

Supplementary Information

## Figures and Tables

**Table 1:** Summary of crop history and N addition as organic amendments or synthetic N fertilizer over the past 12 months.

	Urea fertilized	Organic fertilized	Unfertilized
Crop cycle stage	1^st^ Ratoon crop	Plant crop	1^st^ Ratoon crop
Crop age (weeks)	11	10	11
Organic N addition	~20 kg N ha^−1^ as sugarcane litter (11 weeks)	~80 kg N ha^−1^ as soybean litter (6 months) ~300 kg N ha^−1^ as mill mud (12 months)	~20 kg N ha^−1^ as sugarcane litter (11 weeks)
Synthetic N addition	135 kg N ha^−1^ urea (10 days)	None	None

The quantity of N applied is shown with time since application in parentheses.
