# Supplementary material for: Nitrogen fluxes at the root-soil interface show a mismatch of nitrogen fertilizer supply and sugarcane root uptake capacity
Source: Sci Rep. 2015 Oct 26;5:15727. doi: 10.1038/srep15727 (PMC4620560; doi:10.1038/srep15727)
Supplement: Supplementary Information [file srep15727-s1.pdf]

Online Supplementary Materials

Nitrogen fluxes at the root-soil interface show a mismatch of nitrogen fertilizer supply and root uptake capacity

Authors: Richard Brackin, Torgny Näsholm, Nicole Robinson, Stéphane Guillou, Kerry Vinall, Prakash Lakshmanan, Susanne Schmidt, Erich Inselsbacher

Table S1. Ammonium-N, nitrate-N and amino (AA)-N fluxes and concentrations in the three soils under sugarcane. Numbers in parentheses are standard error of the mean of 40-48 replicate soil extractions or flux measurements. Capital superscript letters represent significance (*P*<0.05) from a Tukey's post-hoc test comparing soil types within each N form and type of N sampling.

	**Diffusive flux** (nmol N cm^{-2} h^{-1})			**Free N** (µmol N kg^{-1} dry soil)			**Exchangeable N** (µmol N kg^{-1} dry soil)		
	**Urea fertilised**	**Organic fertilised**	**Unfertilised**	**Urea fertilised**	**Organic fertilised**	**Unfertilised**	**Urea fertilised**	**Organic fertilised**	**Unfertilised**
**NH_4^+**	3017 (515)^{A}	28 (2)^{B}	4 (0.3)^{C}	9877 (1384)^{A}	366 (27)^{B}	5 (1)^{C}	14246 (1642)^{A}	1534 (140)^{B}	50 (6)^{C}
**NO_3^-**	27 (14)^{B}	68 (4)^{A}	1 (0.2)^{C}	378 (55)^{A}	450 (29)^{A}	13 (1)^{B}	1005 (533)^{A}	407 (27)^{A}	40 (5)^{B}
**AA**	13 (1)^{B}	19 (1.4)^{A}	14 (1.2)^{B}	4 (1)^{A}	2 (0.3)^{A}	5 (0.4)^{A}	51 (3)^{A}	50 (2)^{A}	48 (2)^{A}
**Total LMW-N**	**3057**	**115**	**19**	**10259**	**818**	**23**	**15302**	**1991**	**138**

Table S2. Soil properties of the studied commercial fields under sugarcane. Data represents averages of five replicate soil samples, with standard error of the mean in parentheses.

**Soil and climate**	
Soil type	Hydrosol (Australian Soils Classification)
pH (H_2O)	4.87 (0.1)
Electrical conductivity (µS cm^{-1})	315 (59.6)
Organic carbon (%C)	2.1 (0.1)
Total nitrogen (%N)	0.28 (0.003)
Annual mean maximum temperature (°C)	25.1
Annual mean minimum temperature (°C)	17.2
Annual rainfall (mm)	1300

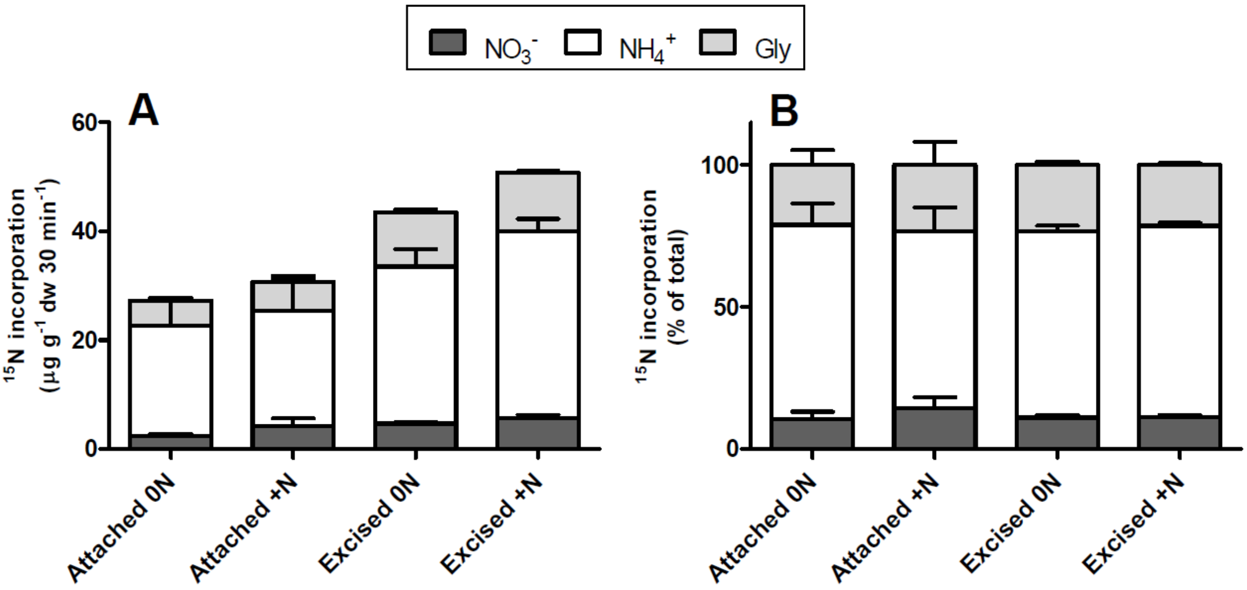

Figure S1. Comparing the uptake of nitrate, glycine and ammonium by attached and excised roots incubated in equimolar solution of the three N sources with one N source ^{15}N -labelled and two N sources unlabelled (1000 µM each N source). Roots were excavated from fertilised (+N) or unfertilised (0N) sugarcane plants in a field trial near Bundaberg, Queensland. The uptake experiment was conducted in summer (January).

Supplementary methods:

Nitrogen source selectivity of attached roots

Experiments with attached roots were carried out to validate or reject results from the excised root experiments at one time point and with one N concentration. The experiment was conducted in summer (23 and 24 January) and the air temperature during the experiment was 30–35°C. Roots, including tips, were excavated by carefully brushing away soil from the soil surface until roots were exposed. The roots used in the *in situ* experiment were young, fleshy, white roots including the tip and elongation zone of the major root axis, as well as branch roots above this region similar to the roots used in the excised roots experiments. Roots were excavated by hand from approximately 0–10 cm depth. Roots were left attached to plants, covered with damp paper towel to keep moist and incubated within 3 h of excavation. For each plant, roots were divided into three groups. Each set of roots was placed in a vial containing a 3000 μM N solution with equimolar concentrations of each of ammonium (as $(NH_4)_2SO_4$), nitrate (as KNO_3) and glycine (1000 μM N each source), with one of the N sources labelled with ^{15}N (98–99 atom%) and the other two N sources at natural abundance level. Roots were incubated for 30 min before being severed just above the vial, rinsed for 10 min with 10 mM KCl, followed by a 1 min rinse with rainwater. Root samples were dried and analysed as described above for excised roots.

^{15}N uptake by attached roots

Attached roots from fertilised and unfertilised plants acquired similar amounts of the three offered N sources (Fig. 3.6). Attached roots incorporated on average 38% less ^{15}N from all N sources than excised roots ($p<0.05$; Fig. 3.5), but proportions of ^{15}N incorporation of the three N sources were similar in attached and excised roots regardless of fertiliser treatment. This suggests the possibility that excised root experiments over-estimate N uptake rates: if this is the case, then I_{max} values would be smaller than those reported here by ~30%. The potentially higher I_{max} values from excised roots which we have used represent a conservative estimate of the mismatch between soil fluxes and root uptake.
